# *Dracaena arborea* alleviates ultra-structural spermatogenic alterations in streptozotocin-induced diabetic rats

**DOI:** 10.1186/1472-6882-13-71

**Published:** 2013-04-02

**Authors:** Modeste Wankeu-Nya, Adrian Florea, Stefana Bâlici, Pierre Watcho, Horea Matei, Albert Kamanyi

**Affiliations:** 1 Department of Cellular and Molecular Biology, “Iuliu Haţieganu” University of Medicine and Pharmacy, 6 L. Pasteur St, Cluj-Napoca 400359, Romania; 2 Animal Physiology and Phytopharmacology Laboratory, University of Dschang, P.O. BOX 67, Dschang, Cameroon

**Keywords:** Streptozotocin, Diabetes, Testes, Spermatogenesis, *Dracaena arborea*, Rats

## Abstract

**Background:**

Infertility is a common complication in diabetic men and experimental animals, mainly due to loss of germ cells by apoptotic cell death. The aim of this study was to evaluate the effects of aqueous and ethanol extracts of *Dracaena arborea* in streptozotocin-induced ultra-structural spermatogenic alterations in Wistar rats.

**Methods:**

Diabetic animals were orally treated with Millipore water (10 ml/kg), sildenafil citrate (1.44 mg/kg) or *Dracaena arborea* aqueous (500 mg/kg) and ethanol (100 mg/kg) extracts for three weeks. A group of non diabetic rats received Millipore water (10 ml/kg) and served as healthy control group. Blood glucose was monitored at the beginning and the end of the study. One day after the last treatment, animals were sacrificed and the testes immediately removed were morphologically observed and prepared for electron microscopy analysis of spermatogenesis.

**Results:**

Our results showed that *Dracaena arborea* was devoid of any anti-hyperglycemic activity. In the untreated diabetic rats, hyperglycemia severely damaged the testes morphology as well as the spermatogenic process as evidenced by the: thickness of basement membrane of the seminiferous tubule; mitochondria alteration; abnormal spermatocyte cells displaying polymorphous nuclei, cytoplasmic vacuolization and necrosis; and disorganization and degeneration of sperm germ cells. Administration of sildenafil citrate and *Dracaena arborea* extracts to the diabetic rats improved testes morphology and reversed, although not completely, the impairment of spermatogenesis; this alleviating effect was more pronounced in animals treated with the aqueous extract (500 mg/kg) of *Dracaena arborea*.

**Conclusion:**

*Dracaena arborea* improves testes morphology and restores spermatogenesis in type 1 diabetic rats, without having major anti-hyperglycemic properties. These effects could be attributed to saponins, flavonoids, phenols and sterols revealed in this plant, which could be a useful component in the treatment of diabetes-induced testicular dysfunction.

## Background

Infertility is one of the major health problems in life, and approximately 30% of this problem is due to male factors. Male infertility is a multifactorial disease, with numerous factors contributing to both reduced spermatogenesis and production of dysfunctional sperm – the most prevalent underlying characteristics found in cases of idiopathic male infertility [1,2]. Infertility is a common complication in diabetic men and experimental animals [3,4], and oxidative stress and peroxidative damage are believed to be the most influential harm causing factors [5,6]. It has been shown that insulin-dependent diabetes mellitus (type 1 diabetes mellitus - DM) in both humans and animals lead to decrease of testicular weight, structural and ultra-structural changes in the spermatogenic process [3,7]. These alterations are mainly due to the loss of germ cells by apoptotic cell death, low testosterone and lack of insulin [8,9]. Understanding the mechanisms underlying type 1 diabetes-induced alterations of spermatogenesis is essential for the development of a strategy to prevent/alleviate them. Type 1 diabetes is a useful tool to study the insulin-related modulation of testicular function. However, since modern treatment options available for the regulation of testicular function anomalies induced by diabetes are becoming more expensive and often carry serious side effects, many people now rely on herbal medicines for health care. Cameroonian traditional medicine indicates that *Dracaena arborea* root extracts have been locally used by many people as aphrodisiac to treat sexual inadequacy and to stimulate sexual vigor. In a pilot study, we demonstrated that the aqueous (500 mg/kg) and ethanol (100 mg/kg) extracts of *Dracaena arborea* stimulate copulatory activity of normal and androgen-deprived (castrated) rats through dopaminergic and/or cholinergic pathways [10].

On the basis of the above mentioned findings, the present study was undertaken to evaluate the effects of the aphrodisiac plant, *Dracaena arborea*, on the ultra-structural spermatogenic damages in streptozotocin-induced type 1 diabetic rats. Data from the literature considered as aphrodisiacs substances that enhance sex drive and/or sexual pleasure or can arouse sexual desire or libido; they are also agents that can be used to modify impaired sexual functions [11-14].

## Methods

### Plant material collection and preparation of extracts

The plant material was harvested in Bagnoun, West Region of Cameroon and authenticated at the Cameroon National herbarium (CNH) under the voucher number 25361/SFR/Cam. The harvested fresh root barks were cut into small pieces, dried at room temperature and ground into a fine powder. For the aqueous extraction, eight hundred grams (800 g) of the powdered roots were dissolved in 5 l of distilled water and kept for 72 h at 25°C, and occasionally stirred. After filtration, the filtrate was oven-concentrated (40°C) to give 39.68 g of brownish residue corresponding to an extraction yield of 4.96%. In order to obtain the ethanolic extract, ground root bark (1 kg) of *Dracaena arborea* was macerated with ethanol (95%) (5 l, 2x) for 72 hours to yield, after solvent evaporation under reduced pressure, 30 g of a brownish extract corresponding to an extraction yield of 3%. The two extracts used in the study were prepared at a final concentration of 100 mg/ml in Millipore water; the volume of oral administration was 1 ml/100 g of body weight.

### Phytochemical screening

Qualitative phytochemical evaluation was performed on aqueous and ethanolic extracts of *Dracaena arborea* to determine the presence of flavonoids (test of Shinoda), sterols (Libermann Buchard test), phenols (ferric chloride test), alkaloids (Dragendorff test) and saponins (Saponification test). All these tests were performed using standard methods [15].

### Experimental animals

Male Wistar rats weighing 200-250 g, grown in the Animal House of “Iuliu Haţieganu” University of Medicine and Pharmacy, Cluj-Napoca, Romania, were acclimatized for one-week in the Department of Cellular and Molecular Biology, of the above mentioned University. They were placed in Plexiglas cages under a 12-hour light: dark cycle with standard food and water ad libitum. All experiments were approved by the ethical committee of the University of Medicine and Pharmacy, Cluj-Napoca, Romania through letter Nr 687A/30.10.2012.

### Induction of diabetes

Experimental diabetes was induced in overnight fasted rats by a single dose of streptozotocin (STZ) (50 mg/kg) dissolved in ice-cold 0.1 M sodium citrate buffer, pH 4.5 and administered i.p. One hour-thirty minutes after injection of STZ, all animals received an intraperitoneal injection of glucose 33% to overcome hypoglycemia (fatal in rats) induced by STZ after the pancreatic β-cell destruction and massive release of insulin. 48 h after STZ treatment, the fasting blood glucose level was measured using reagent strips (Accu-Chek^®^, Roche) with a drop of blood obtained by tail-vein puncture. Animals were considered diabetic if blood glucose values were higher than 200 mg/dl.

### Animal groups

Two weeks after the confirmation of the diabetic state, animals were randomly divided into 5 groups of 5 animals each and treated as follows: Group 1, healthy rats receiving 10 ml/kg of Millipore water only (Healthy control group); Group 2, diabetic rats receiving 10 ml/kg of Millipore water only (Diabetic control group); Group 3, diabetic rats treated with 1.44 mg/kg of sildenafil citrate; Group 4, diabetic rats treated with the aqueous extract of *Dracaena arborea* (500 mg/kg); Group 5, diabetic rats treated with the ethanolic extract of *Dracaena arborea* (100 mg/kg). The vehicle (Millipore water) and the test solutions were orally administered once a day for 3 weeks using an endogastric canule. Doses of plant extracts were chosen on the basis of our pilot studies [10]. Initial and final blood glucose measurements as well as gross testes morphology were performed in this study.

### Drugs

Streptozotocin (Sigma-Aldrich N.V/S.A. K. Cardijnplein 8, B-2880 BORNEM) and sildenafil citrate (Pfizer) (Cluj-Napoca, Romania) were used in this study.

### Statistical analysis

In each treatment group, the effect of treatment duration on blood glucose level was analysed using two ways ANOVA repeated measures followed by Bonferroni all pair comparison test. However, two ways ANOVA followed by Bonferroni all pair comparison test was used to compare the treated groups to healthy and diabetic control animals respectively. P values of 0.05 or less were taken to imply statistical significance between the means. All these analysis were performed using Graphpad Prism version 5.1.

### Experimental procedure for electron microscope observation

Immediately after the sacrifice of rats under chloroform anesthesia, 3 small pieces from each testis were collected and prefixed with 2.7% glutaraldehyde in 0.1 M phosphate saline buffer for 2 hours. After being washed 4 times in the same buffer (the first 3 for 1 hour and the last, overnight), samples were post-fixed with 1% osmium tetroxide in 0.15 M phosphate saline buffer for 2 hours, and washed again 2 times (30 min each) in the same buffer. The samples were next dehydrated in acetone solutions (prepared in distilled water), once in 30%, 50%, 70%, 80%, 90% and three times in 100% for 15 min respectively except that of 50% which was done for 30 min. All the steps from the beginning up to 70% acetone were performed at 4°C while the others (from 80 to 100%) were done at room temperature. After complete dehydration, the samples were infiltrated with Epon 812 in 4 steps: acetone-epon 2:1 (45 min), acetone-epon 1:1 (1 hour), acetone-epon 1:2 (1 hour), pure epon (overnight). The samples were then placed in gelatin capsules with fresh epon, and polymerized at 60°C for 72 hours. Trapezoidal ultrathin sections of 70–80 nm thickness were obtained from 3 seminiferous tubules of 4 blocks from each group with glass knives using a Bromma 8800 ULTRATOME III (LKB, Sweden) and contrasted next with solutions of uranyl acetate (15 min) and lead citrate (8 min). The examination of these sections was performed on a Jeol JEM 1010 transmission electron microscope (Jeol, Tokyo, Japan). Images were captured using a Mega VIEW III camera (Olympus, Soft Imaging System, Munster, Germany), and introduced in a database using a Soft Imaging System software (Soft Imaging System, Munster, Germany). Ultra-structural features of the prepared seminiferous tubule section of each group were then evaluated.

## Results

### Phytochemical analysis

Qualitative phytochemical screening of both aqueous and ethanol extracts of *Dracaena arborea* dried roots showed the presence of saponins, phenols, flavonoids and sterols.

### Effect of treatments on the diabetic rat testis morphology

**Figure 1:**
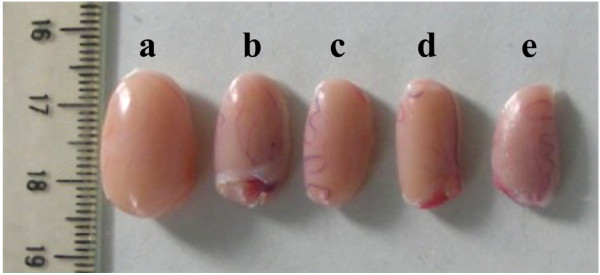
**Photograph showing from left to right testes of rat in healthy control group (a) and diabetic rats treated with aqueous extract (b), ethanol extract (c) of *Dracaena arborea*, sildenafil citrate (d) and Millipore water (e) for three weeks.**

Injection of streptozotocin to rats resulted in a severe atrophy of the testes (Figure 1e) compared to healthy rats receiving Millipore water (Figure 1a). The treatment of animals with either plant extracts (Figure 1b, c) or sildenafil citrate (Figure 1d) for three weeks partially corrected the impaired morphology of the testes.

### Effect of treatments on fasting blood glucose level

Data from Table 1 clearly showed the detrimental effect of streptozotocin on blood glucose levels. In all diabetic animals, a huge rise in the final blood glucose concentrations was noticed. Thus, an increase of 60.34% was recorded in the untreated diabetic group compared to the aqueous (15.39% of increase), ethanolic (19.04% of increase) extracts of *Dracaena arborea* and sildenafil citrate (23.59% of variation). These results further revealed the lack of effect of *Dracaena arborea* extracts and sildenafil citrate in reducing blood glucose in 3 weeks treated-diabetic rats.

**Table 1:** Variation of blood glucose levels in rats after different treatments

	**Blood glucose level (mg/dl)**		
**Groups**	**At the beginning of the treatment (Initial)**	**At the end of the treatment (Final)**	**% of variation**
Healthy control (Millipore water, 10 ml/kg)	98.40 ± 02.39^a^	99.20 ± 01.02^a^	0.81
Diabetic control (Millipore water, 10 ml/kg)	374.20 ± 31.82^a***^	>600 ± 00.00^d***^	>60.34
Treatment 1 (Sildenafil citrate 1.44 mg/kg)	371.40 ± 23.81^a***^	459.00 ± 19.60^cγ***^	23.59
Treatment 2 (*D.a*. aqueous extract 500 mg/kg)	367.80 ± 21.70^a***^	424.40 ± 26.71^cγ***^	15.39
Treatment 3 (*D.a.* ethanol extract 100 mg/kg)	366.60 ± 08.66^a***^	436.40 ± 25.94^cγ***^	19.04

*D.a.* = *Dracaena arborea*. Values are means ± SEM, number of animals per group = 5. On the same line, ANOVA two ways repeated measures followed by Bonferroni all pair comparison test was done; the values assigned by the same letter are not significantly different; ^d ^p < 0.001; ^c ^p < 0.01 compared to the initial blood glucose level. In the same column, two ways ANOVA followed by Bonferroni all pair comparison test was performed; *** p <0.001 compared to the corresponding normal control; ^γ ^p <0.001 significantly different compared to the corresponding diabetic control. Variation of blood glucose level (%) = (final – initial) × 100/initial.

### Electron microscope observations of the effects of different treatments on spermatogenesis

#### Healthy control group

**Figure 2:**
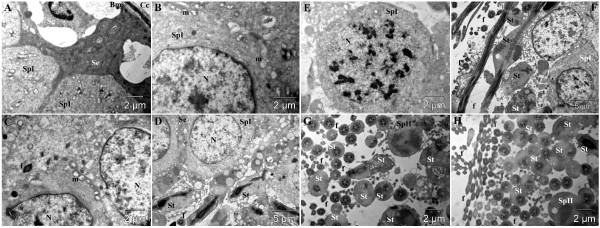
**Ultra-structural micrograph of seminiferous tubule portion of healthy control rats treated with Millipore water (10 ml/kg), showing normal spermatogenic parameters. **Basement membrane (**Bm**) of the seminiferous tubule; cell of lamellated connective tissue (**Cc**); spermatogonium (**Sg**); Sertoli cell (**Se**); primary spermatocyte (**SpI**); secondary spermatocyte (**SpII**); spermatid (**St**); nucleus (**N**); mitochondria (**m**); flagellae of sperm cells (**f**).

In the healthy control group, a normal aspect of the basement membrane with a normal diameter was observed. A normal space between this basement membrane and spermatogonia and spermatocyte was also observed (Figure 2A). Spermatocytes appeared with normal aspect of nuclei, plasma membrane and cytoplasm. No apoptotic and necrotic cells were noted in this group. Mitochondria, Sertoli cells and spermatogonia have normal appearance (Figure 2B-D). Spermatogenesis process was normal and characterized by a high number of spermatocyte I, spermatocyte II, spermatids and sperm cells (Figure 2E-H).

### Diabetic control group

**Figure 3:**
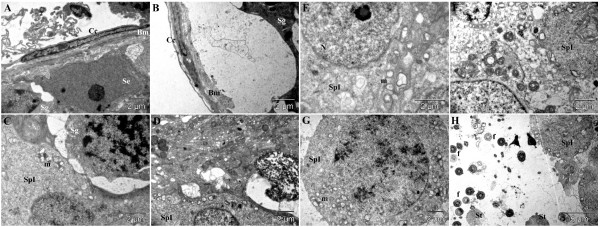
**Ultra-structural micrograph of seminiferous tubule portion in diabetic control rats treated with Millipore water (10 ml/kg) showing many apoptosis, necrosis and abnormal spermatogenic cells. **Basement membrane (**Bm**) of the seminiferous tubule; cell of lamellated connective tissue (**Cc**); spermatogonium (**Sg**); Sertoli cell (**Se**); primary spermatocyte (**SpI**); secondary spermatocyte (**SpII**); spermatid (**St**); nucleus (**N**); mitochondria (**m**); flagellae of sperm cells (**f**).

In the untreated diabetic group, a thickening of the basement membrane was detected (Figure 3A, B), coupled to larger spaces between basement membrane and spermatogonia in many areas (Figure 3B). Many apoptotic primary spermatocytes were observed (Figure 3D), displaying shrunken, polymorphous nuclei and cytoplasmic vacuolization. In many primary spermatocytes, cytoplasmic vacuoles were also observed, especially around modified mitochondria (Figure 3E); other primary spermatocytes appeared to be in a necrotic state with normal aspect of nuclei but with disrupted plasma membrane, and rarefied cytoplasm (Figure 3F). The layer of primary spermatocytes was very thin as compared to the healthy control group, only some of them undergoing meiosis (Figure 3G). The secondary spermatocytes and spermatids were rare and a very low number of sperm cells was noted (Figure 3H).

### Treatment group 1 (sildenafil citrate 1.44 mg/kg)

**Figure 4:**
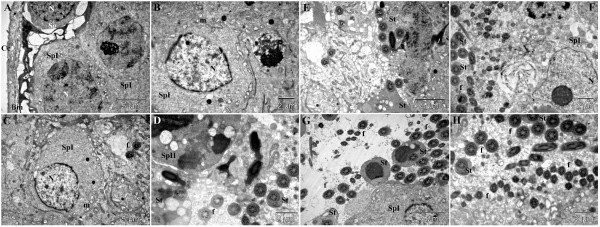
**Ultra-structural micrograph of seminiferous tubule portion of diabetic rats treated with sildenafil citrate (1.44 mg/kg). **Basement membrane (**Bm**) of the seminiferous tubule; cell of lamellated connective tissue (**Cc**); spermatogonium (**Sg**); Sertoli cell (**Se**); primary spermatocyte (**SpI**); secondary spermatocyte (**SpII**); spermatid (**St**); nucleus (**N**); mitochondria (**m**); flagellae of sperm cells (**f**).

As compared to healthy control group, no important changes concerning the aspect of basement membrane were observed in this group (Figure 4A). Even if some small empty spaces between the basement membrane and the spermatogonia and primary spermatocytes were observed, they seem to be normal (Figure 4A). Apoptotic cells were also observed in this group (Figure 4B), but in lower number as compared to untreated diabetic group. Primary spermatocytes were present in high number, and even if a small number of secondary spermatocytes were observed, spermatogenesis occurred normally, marked by a higher number of spermatids and sperm cells as compared to untreated diabetics (Figure 4D-H).

### Treatment group 2 (***Dracaena arborea***, 500 mg/kg aqueous extract)

**Figure 5:**
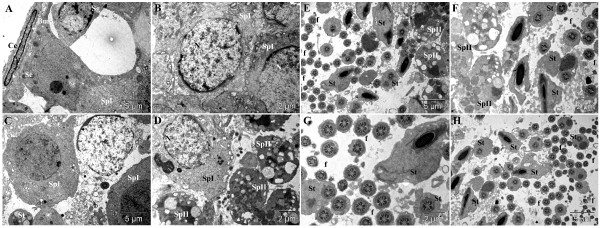
**Ultra-structural micrograph of seminiferous tubule portion of diabetic rats treated with aqueous extract of *Dracaena arborea *(500 mg/kg). **Observations from this group are similar and even most important compared to the heahthy control group. Basement membrane (**Bm**) of the seminiferous tubule; cell of lamellated connective tissue (**Cc**); spermatogonium (**Sg**); Sertoli cell (**Se**); primary spermatocyte (**SpI**); secondary spermatocyte (**SpII**); spermatid (**St**); nucleus (**N**); mitochondria (**m**); flagellae of sperm cells (**f**).

The electron microscopic images of this group were comparable to those of the healthy control group, proving the effectiveness of the applied treatment. The aspect of the basement membrane joined to the appearances of Sertoli cells and spermatogonia were similar to that observed in the healthy control group (Figure 5A). The primary spermatocytes had a normal aspect (Figure 5A-D), even if some rare necrotic cells were noted (Figure 5C). Also, secondary spermatocytes (Figure 5D-F) and spermatids (Figure 5C-H) were present in high numbers. In this group, a very high number of sperm cells, even higher than in the healthy control group were observed in different regions of many tubules (Figure 5E-H).

### Treatment group 3 (***Dracaena arborea***, 100 mg/kg ethanol extract)

**Figure 6:**
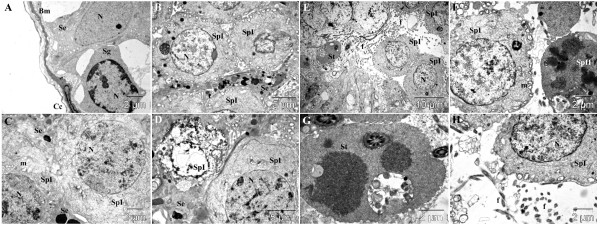
**Ultra-structural micrograph of seminiferous tubule portion of diabetic rats treated with ethanol extract of *Dracaena arborea *(100 mg/kg). **Observations from this group are similar to those of group 3. Basement membrane (**Bm**) of the seminiferous tubule; cell of lamellated connective tissue (**Cc**); spermatogonium (**Sg**); Sertoli cell (**Se**); primary spermatocyte (**SpI**); secondary spermatocyte (**SpII**); spermatid (**St**); nucleus (**N**); mitochondria (**m**); flagellae of sperm cells (**f**).

The obtained results in this group can be placed under those obtained for diabetic rats treated with sildenafil citrate. The basement membrane and spermatogonia had a normal aspect (Figure 6A), but most of the Sertoli cells were loaded with inclusions (Figure 6B-D), and so were some of the primary spermatocytes (Figure 6B). Many primary spermatocytes were observed in a necrotic state (Figure 6D). Rare secondary spermatocytes were found displaying vacuolizations similar to those observed in autophagy processes (Figure 6F,G). Also, very rare spermatids were noted (Figure 6E), and a relative low number of sperm cells as well (Figure 6A, D).

## Discussion

In many animal species, STZ induces diabetes that resembles human hyperglycemic nonketotic diabetes mellitus and this effect has been extensively studied and appears to be mediated through a lowering of beta cell nicotinamide adenine dinucleotide (NAD+) and results in histopathologic alteration of pancreatic islet beta cells [16,17]. It is known that the normal spermatogenesis process takes about 52–53 days in rat against 74 in human. The development of the seminiferous epithelium cycle or the spermatogenetic cycle is segmental in rat, has 14 stages and takes place in 287.7 hours (12 days), while in human it is helical, has 6 stages and occurs in 384.6 hours (16 days) [18].It has been demonstrated that, one or more stages of this spermatogenic process can be altered during diabetes [3,7,19]. In the present study, we found that STZ-induced diabetes in rats caused testicular dysfunction, leading to the dramatic changes in the testicular morphology and the alteration of spermatogenic process. This atrophy of the testes is mainly due to the decrease in testosterone level associated with the absence or diminution of serum insulin levels, since insulin acts as an anti-apoptotic factor capable to regulate testicular apoptosis and sexual dysfunction induced by diabetes [20-22]. Spermatogenesis dysfunction in the diabetic control group was revealed in electron micrograph examinations by the atrophy of the seminiferous tubules characterized by: the decreased tubule diameters and the thickness of the basement membrane diameter of the seminiferous tubule; the reduction of the spermatogenic cell series; the modified mitochondria; the disorganization and degeneration of spermatocytes, spermatids and sperm germ cells. Seminiferous tubule atrophy and decrease in spermatogenic cells are important indicators of spermatogenesis failure. Moreover, diabetes increases thickening of the seminiferous tubules basement membrane and this thickness is accompanied by a decreased rate of sperm production and an overall reduction in the size or diameter of seminiferous tubules [23-25]. All these abnormal phenomena observed in this study could be attributed to the toxic effect of STZ on male fertility as streptozotocin adversely affects fertility when administered to male rats, through disruption of testicular function caused by a decrease in testosterone levels [26,27].

Testicular tissue damage caused by STZ-induced diabetes in rat as observed in this study, could then be responsible for the reduction and the death of germ cells [3,4,27]. In mammals, the mechanism of action that results in cell death has not been fully identified; however, it is thought to be a result of DNA and chromosomal damage brought forth by mechanisms involving free radicals generation during STZ metabolism, resulting in cell death by apoptosis or necrosis, by increasing the expression of cytochrome c and caspases 9 and 3, which in turn result in a high frequency of single and double stranded DNA breaks [21,26,28]. Therefore, free radicals production resulting in oxidative stress is a popular theory that explains the etiology and pathophysiology of the biological effects of diabetes mellitus, especially in regard to cell damage, cellular degeneration, and subsequent complications [29].

However, when compared to the untreated diabetic group, three weeks of oral administration of sildenafil citrate or *Dracaena arborea* extracts to diabetic rats alleviated, although not completely, these ultra-structural deficits by providing protection against apoptotic germ cell death and impairment in the spermatogenic process. *Dracaena arborea* could then act like sulphurous mineral water and sodium hydrosulphide on diabetic rat testes [30]. These reversal effects observed in the treated rats, especially in the aqueous plant extract-treated animals, were similar to those of the healthy control group. These alleviating effects of *Dracaena arborea* extracts in testicular damages of STZ diabetic rats are similar to those obtained after application of hydrated C60 fullerene or enalapril in STZ-induced diabetic rats [31,32]. Since oxidative stress and low androgenic hormone levels are the main causing factors of testicular dysfunction and impairment of spermatogenesis in diabetic rats [7,33], the mechanism(s) of alleviation of the testicular lesions seen in diabetic rats orally given the extracts of *Dracaena arborea*, may include the antioxidant [34] and androgenic effects of flavonoids [35], phenols [36], saponins [37,38] and phytosterols [39] revealed in the aqueous and ethanolic extracts of *Dracaena arborea*. The androgenic hypothesis could be confirmed by the sexual stimulant effects of the aqueous and ethanolic extracts of this plant in the copulatory activity of androgen-deprived (castrated) rats as previously demonstrated by our research group [10]. The regulatory effect of sildenafil citrate in spermatogenic alteration observed in the present study may firstly be attributed to its steroidogenic activity since it was demonstrated that, inhibition of PDE activity during prolonged sildenafil treatment increased serum testosterone level and Leydig cells’ steroidogenic capacity by coordinated stimulatory action on cAMP and cGMP signaling pathway [40]. Then, sildenafil citrate may have some neurophysiological potential on male fertility profile, through the normalization of spermatogenesis and the improvement of the entire smooth musculature of reproductive tract as well as testicular morphology altered by neuropathic diabetes condition (by reducing the excess accumulation of interstitial collagen and calcification in the smooth muscles of seminiferous tubules which made them rigid leading to a decreased sperm motility) [41]. Also, sildenafil citrate administration may have cytoprotective effects by reducing oxidative stress and apoptosis [42,43].

## Conclusion

Our results clearly demonstrate that the increased rate of testicular germ cell death by apoptosis in streptozotocin-induced diabetic rats was protected and regulated by the administration of sildenafil citrate and *Dracaena arborea* especially the aqueous extract (500 mg/kg), without having major anti-hyperglycemic property. These alleviating effects could be attributed to saponins, flavonoids and sterols present in this plant, which could be a useful treatment in preventing and treating diabetes-induced testicular dysfunction.

## Competing interests

The authors declare that they have no competing interests.

## Authors’ contributions

MWN, PW, SB, AF and HM conceived the project. MWN and SB did the literature search. MWN, SB and AF contributed to the laboratory work. AF did the electron micrographic images with their interpretations. HM and AK was responsible for the overall supervision. MWN and PW wrote the paper with input from all the authors. All authors read and approved the final manuscript.

## Pre-publication history

The pre-publication history for this paper can be accessed here:

http://www.biomedcentral.com/1472-6882/13/71/prepub

## References

[B1] Tambi MI Imran MK Eurycoma longifolia jack in managing idiopathic male infertility Asian J Androl 2010 12 Suppl 3 376 380 20348942 10.1038/aja.2010.7 PMC3739276

[B2] Cao JF Zhang PY Xu CW Huang TT Bai YG Chen KS **Effect of aqueous extract of *****Arctium lappa *****L. (Burdock) roots on the sexual behavior of male rats.** BMC Complement Altern Med 2012 12 8 10.1186/1472-6882-12-8 22296876 PMC3299611

[B3] Ricci G Catizone A Esposito R Pisanti FA Vietri MT Galdieri M Diabetic rat testes: morphological and functional alterations Andrologia 2009 41 361 368 10.1111/j.1439-0272.2009.00937.x 19891634

[B4] Mallidis C Agbaje I McClure N Kliesch S The influence of diabetes mellitus on male reproductive function: a poorly investigated aspect of male infertility Urology A 2011 50 33 37 10.1007/s00120-010-2440-3 21207007

[B5] Boujbiha MA Hamden K Guermazi F Bouslama A Omezzine A Kammoun A Feki AE Testicular toxicity in mercuric chloride treated rats: association with oxidative stress Reprod Toxicol 2009 28 81 89 10.1016/j.reprotox.2009.03.011 19427169

[B6] Mohasseb M Ebied S Yehia MA Hussein N Testicular oxidative damage and role of combined antioxidant supplementation in experimental diabetic rats J Physiol Biochem 2011 67 185 194 10.1007/s13105-010-0062-2 21184211

[B7] La Vignera S Condorelli R Vicari E D’agata R Calogero AE Diabetes mellitus and sperm parameters J Androl 2012 33 Suppl 2 145 153 21474785 10.2164/jandrol.111.013193

[B8] Zhao H Xuc S Wang Z Li Y Guo W Lina C Gong S Li C Wange G Caie L Repetitive exposures to low-dose X-rays attenuate testicular apoptotic cell death in Streptozotocin-induced diabetes rats Toxicol Lett 2010 192 356 364 10.1016/j.toxlet.2009.11.011 19931367

[B9] Zhao Y Tan Y Dai J Li B Guo L Cui J Wang G Shi X Zhang X Mellen N Li W Cai L Exacerbation of diabetes-induced testicular apoptosis by zinc deficiency is most likely associated with oxidative stress, p38 MAPK activation, and p53 activation in mice Toxicol Lett 2011 200 100 106 10.1016/j.toxlet.2010.11.001 21078376

[B10] Watcho P Wankeu-Nya M NGuelefack TB Tapondjou L Teponno R Kamanyi A **Pro-sexual effects of *****Dracaena arborea *****(willd) link (Dracaenaceae) in sexually experienced male rats.** Pharmacologyonline 2007 1 400 419

[B11] Sandroni P Aphrodisiacs past and present: a historical review Clin Auton Res 2001 11 Suppl 5 303 307 11758796 10.1007/BF02332975

[B12] Yakubu MT Akanji MA Oladiji AT **Aphrodisiac potentials of the aqueous extract of *****Fadogia agrestis *****(schweinf. Ex hiern) stem in male albino rats.** Asian J Androl 2005 7 Suppl 4 399 404 16281088 10.1111/j.1745-7262.2005.00052.x

[B13] Yakubu MT Afolayan AJ Effect of aqueous extract of *Bulbine natalensis* (Baker) stem on the sexual behaviour of male rats Int J Androl 2009 32 Suppl 6 629 636 18710410 10.1111/j.1365-2605.2008.00910.x

[B14] Shamloul R Natural aphrodisiacs J Sex Med 2010 7 39 49 10.1111/j.1743-6109.2009.01521.x 19796015

[B15] De S Dey YN Ghosh AK Phytochemical investigation and chromatographic evaluation of the different extracts of tuber of *amorphaphallus paeoniifolius* (araceae) Int J Pharm Biomed Res 2010 1 Suppl 5 150 157

[B16] Bolzan AD Bianchi MS Genotoxicity of Streptozotocin Mutat Res 2002 512 121 134 10.1016/S1383-5742(02)00044-3 12464347

[B17] Brenna O Qvigstad G Brenna E Waldum HL Cytotoxicity of Streptozotocin on neuroendocrine cells of pancreas and the gut Dig Dis Sci 2003 48 906 910 10.1023/A:1023043411483 12772787

[B18] Hess RA De Franca LR Chen CY Spermatogenesis and cycle of the semineferous epithelium Molecular mechanism in spermatogenesis. Volume 636 2008 Texas: Landes Bioscience and Springer Science + Business Media LLC 1 15

[B19] Mallidis C Agbaje I O’Neill J McClure N The influence of type 1 diabetes mellitus on spermatogenic gene expression Fertil Steril 2009 92 Suppl 6 2085 2087 19589518 10.1016/j.fertnstert.2009.06.006

[B20] Ballester J Muñoz MC Domínguez J Rigau T Guinovart JJ Rodríguez-Gil JE Insulin-dependent diabetes affects testicular function by FSH- and LH-linked mechanisms J Androl 2004 25 Suppl 5 706 719 15292100 10.1002/j.1939-4640.2004.tb02845.x

[B21] Cai L Chen S Evans T Deng DX Mukherjee K Chakrabarti S Apoptotic germ-cell death and testicular damage in experimental diabetes: prevention by endothelin antagonism Urol Res 2000 28 Suppl 5 342 347 11127715 10.1007/s002400000134

[B22] Schoeller EL Albanna G Frolova AI Moley KH Insulin rescues impaired spermatogenesis via the hypothalamic-pituitary-gonadal axis in Akita diabetic mice and restores male fertility Diabetes 2012 61 Suppl 7 1869 1878 22522616 10.2337/db11-1527 PMC3379646

[B23] Cameron DF Murray FT Drylie DD Interstitial compartment pathology and spermatogenic disruption in testes from impotent diabetic men Anat Rec 1985 213 53 62 10.1002/ar.1092130108 4073561

[B24] Rohrbach DH Martin GR Structure of basement membrane in normal and diabetic tissue Ann NY Acad Sci 1982 401 203 211 10.1111/j.1749-6632.1982.tb25719.x 6963121

[B25] Sainio-Pollanen S Henriksen K Parvinen M Simell O Pollanen P Stage-specific degeneration of germ cells in the seminiferous tubules of non-obese diabetic mice Int J Androl 1997 20 243 253 10.1046/j.1365-2605.1997.00061.x 9401828

[B26] Amaral S Oliveira PJ Ramalho-Santos J Diabetes and the impairment of reproductive function: possible role of mitochondria and reactive oxygen species Curr Diabetes Rev 2008 4 46 54 10.2174/157339908783502398 18220695

[B27] Koh PO Streptozotocin-induced diabetes increases the interaction of Bad/Bcl-XL and decreases the binding of pBad/14-3-3 in rat testis Life Sci 2007 81 1079 1084 10.1016/j.lfs.2007.08.017 17870134

[B28] Agarwal A Said TM Oxidative stress, DNA damage and apoptosis in male infertility: a clinical approach BJU Int 2005 95 Suppl 4 503 507 15705068 10.1111/j.1464-410X.2005.05328.x

[B29] Ahmed RG The physiological and biochemical effects of diabetes on the balance between oxidative stress and anti-oxidant defense system Med J Islamic World Acad Sci 2005 15 31 42

[B30] Sadik NAH El-Seweidy MM Shaker OG The antiapoptotic effects of sulphurous mineral water and sodium hydrosulphide on diabetic Rat testes Cell Physiol Biochem 2011 28 887 898 10.1159/000335803 22178941

[B31] Bal R Türk G Tuzcu M Yilmaz O Ozercan I Kuloglu T Gür S Nedzvetsky VS Tykhomyrov AA Andrievsky GV Baydas G Naziroglu M Protective effects of nanostructures of hydrated C60 fullerene on reproductive function in Streptozotocin-diabetic male rats Toxicology 2011 282 69 81 10.1016/j.tox.2010.12.003 21163323

[B32] Kushwaha S Jena GB Enalapril reduces germ cell toxicity in Streptozotocin-induced diabetic rat: investigation on possible mechanisms Naunyn-Schmiedeberg's Arch Pharmacol 2012 385 111 124 10.1007/s00210-011-0707-x 22071577

[B33] Chandrashekar KN Muralidhara Evidence of oxidative stress and mitochondrial dysfunctions in the testis of prepubertal diabetic rats Int J Impot Res 2009 21 198 206 10.1038/ijir.2009.9 19444988

[B34] Mallick C Mandal S Barik B Bhattacharya A Ghosh D Protection of testicular dysfunctions by MTEC, a formulated herbal drug, in Streptozotocin induced diabetic rat Biol Pharm Bull 2007 30 84 90 10.1248/bpb.30.84 17202665

[B35] Khaki A Fathiazad F Nouri M Khaki AA Maleki NA Khamnei HJ Ahmadi P Beneficial effects of quercetin on sperm parameters in Streptozotocin-induced diabetic male rats Phytother Res 2010 24 1285 1291 10.1002/ptr.3100 20127875

[B36] Chaiyasut C Kusirisin W Lailerd N Lerttrakarnnon P Suttajit M Srichairatanakool S Effects of Phenolic compounds of fermented Thai indigenous plants on oxidative stress in Streptozotocin-induced diabetic rats Evid Based Complement Alternat Med 2011 2011 10 10.1155/2011/749307 PMC3057567 21423638

[B37] Gauthaman K Adaikan PG Prasad RN **Aphrodisiac properties of *****Tribulus terrestris *****extract (protodioscin) in normal and castrated rats.** Life Sci 2002 71 1385 1396 10.1016/S0024-3205(02)01858-1 12127159

[B38] Kougan GB Miyamoto T Tanaka C Paululat T Mirjolet JF Duchamp O Sondengam BL Lacaille-Dubois MA Steroidal saponins from two species of *dracaena* J Nat Prod 2010 73 Suppl 7 1266 1270 20553003 10.1021/np100153m

[B39] Gupta R Sharma AK Dobhal MP Sharma MC Gupta RS Antidiabetic and antioxidant potential of β-sitosterol in Streptozotocin-induced experimental hyperglycemia J Diabetes 2011 3 29 37 10.1111/j.1753-0407.2010.00107.x 21143769

[B40] Andric SA Janjic MM Stojkov NJ Kostic TS Sildenafil treatment in vivo stimulates Leydig cell steroidogenesis via the cAMP/cGMP signaling pathway Am J Physiol Endocrinol Metab 2010 299 544 550 10.1152/ajpendo.00337.2010 20663985

[B41] Ali ST Rakkah NI Neurophysiological role of sildenafil citrate (sildenafil citrate) on seminal parameters in diabetic males with and without neuropathy Pak J Pharm Sci 2007 20 Suppl 1 36 42 17337426

[B42] Beheshtian A Salmasi AH Payabvash S Kiumehr S Ghazinezami B Rahimpour S Tavangar SM Dehpour AR Protective effects of sildenafil administration on testicular torsion/detorsion damage in rats World J Urol 2008 26 Suppl 2 197 202 18265987 10.1007/s00345-008-0243-6

[B43] Mostafa T Rashed LA Kotb K Testosterone and chronic sildenafil/tadalafil anti-apoptotic role in aged diabetic rats Int J Impot Res 2010 22 Suppl 4 255 261 20574430 10.1038/ijir.2010.12

